# Adipolin/C1q/Tnf-related protein 12 prevents adverse cardiac remodeling after myocardial infarction

**DOI:** 10.1371/journal.pone.0243483

**Published:** 2020-12-04

**Authors:** Tomonobu Takikawa, Koji Ohashi, Hayato Ogawa, Naoya Otaka, Hiroshi Kawanishi, Lixin Fang, Yuta Ozaki, Shunsuke Eguchi, Minako Tatsumi, Mikito Takefuji, Toyoaki Murohara, Noriyuki Ouchi

**Affiliations:** 1 Department of Cardiology, Nagoya University Graduate School of Medicine, Nagoya, Japan; 2 Department of Molecular Medicine and Cardiology, Nagoya University Graduate School of Medicine, Nagoya, Japan; Osaka University Graduate School of Medicine, JAPAN

## Abstract

**Background:**

Myocardial infarction (MI) is a leading cause of death worldwide. We previously identified adipolin, also known as C1q/Tnf-related protein 12, as an anti-inflammatory adipokine with protective features against metabolic and vascular disorders. Here, we investigated the effect of adipolin on myocardial remodeling in a mouse model of MI.

**Methods:**

Male adipolin-knockout (APL-KO) and wild-type (WT) mice were subjected to the permanent ligation of the left anterior descending coronary artery to create MI.

**Results:**

APL-KO mice exhibited increased ratios of heart weight/body weight and lung weight/body weight after MI compared with WT mice. APL-KO mice showed increased left ventricular diastolic diameter and decreased fractional shortening after MI compared with WT mice. APL-KO mice exhibited increased expression of pro-inflammatory mediators and enhanced cardiomyocyte apoptosis in the post-MI hearts compared with WT mice. Systemic administration of adenoviral vectors expressing adipolin to WT mice after MI surgery improved left ventricular contractile dysfunction and reduced cardiac expression of pro-inflammatory genes. Treatment of cultured cardiomyocytes with adipolin protein reduced lipopolysaccharide-induced expression of pro-inflammatory mediators and hypoxia-induced apoptosis. Treatment with adipolin protein increased Akt phosphorylation in cardiomyocytes. Inhibition of PI3 kinase/Akt signaling reversed the anti-inflammatory and anti-apoptotic effects of adipolin in cardiomyocytes.

**Conclusion:**

Our data indicate that adipolin ameliorates pathological remodeling of myocardium after MI, at least in part, by its ability to reduce myocardial inflammatory response and apoptosis.

## Introduction

Ischemic heart disease is one of the major causes of death worldwide [1]. Chronic heart failure after myocardial infarction (MI) is a serious complication which leads to poor prognosis [1–3]. Pathological remodeling of the myocardium, including exacerbation of cardiac dysfunction, myocardial inflammation and cardiomyocyte death causes the progression of heart failure after MI [4]. Thus, it is crucial to establish therapeutic approaches to reduce pathological cardiac remodeling when treating patients with MI [4,5].

Obesity is closely linked to the development of heart failure [6,7]. It is well established that dysregulation of adipose tissue-derived adipokines participates in the development of obese complications. Most of the adipokines, including tumor necrosis factor (TNF) α and interleukin (IL) 6 are pro-inflammatory and promote obesity-related diseases [8–10]. In contrast, a small numbers of adipokines including adiponectin are anti-inflammatory and protect against the development of obese complications [8–10]. In this regard, it has been suggested that some adipokines such as leptin and adiponectin are associated with the progression of heart failure [11–13].

Previously we identified adipolin/C1q/Tnf-related protein 12 as an adipokine that is down-regulated in rodent models of obesity [14]. We found that adipolin improves insulin sensitivity in obese mice by reducing adipose tissue inflammation [14]. Adipolin also ameliorates glucose metabolism through insulin dependent and independent mechanisms [15]. Moreover, we have shown that adipolin attenuates pathological vascular remodeling after artery injury through inhibition of smooth muscle cell proliferation and macrophage inflammatory response [16]. Thus, it is likely that adipolin acts as an anti-inflammatory adipokine that can prevent the development of metabolic and vascular disorders. However, little is known about the role of adipolin in obesity-related heart disease. We proposed that adipolin could affect the development of ischemic heart disease in a paracrine or endocrine manner. Here, we investigated the effect of adipolin on cardiac remodeling in a mouse model of MI using loss-of-function genetic manipulations.

## Materials and methods

### Ethics statement

All animal study protocols were approved by the Institutional Animal Care and Use Committee in Nagoya University and RIKEN Kobe Branch. Our study conformed to the Guide for the Care and Use of Laboratory Animals, published by the United States National Institutes of Health (NIH Publication, 8^th^ Edition, 2011).

### Materials

Antibodies against phospho-Akt (Ser-473), Akt and α-tubulin were purchased from Cell Signaling Technology (Danvers, MA, USA). The polyclonal antibody against mouse adipolin was generated as described previously [17]. LY294002 was purchased from Wako. Lipopolysaccharide (LPS) was purchased from Sigma. Adenoviral vectors expressing the gene for β-galactosidase (Ad-β-gal) and FLAG-tagged adipolin (Ad-APL) were prepared as previously described [14,16].

### Mouse models of myocardial infarction

Adipolin knockout (APL-KO) mice were generated as previously described (Accession No. CDB1102K Website: http://www2.clst.riken.jp/arg/mutant%20mice%20list.html) [16]. Homozygous APL-KO and wild-type (WT) mice with a C57BL/6J background at 10 weeks of age were used in this study. Genotyping primers for the adipolin (Fam 132a/CTRP12) WT allele were as follows: 5’-GCCTGAATCCCCCACTAACT-3’ (P1) and 5’-TCTGGTAGCCCTGAGAATCG-3’ (P2). Primers for the adipolin (Fam 132α/CTRP12) null allele were as follows: 5’-GGAAGTGCCCAATGAGTCC-3’ (P3) and 5’-GTGGATGTGGAAATGTGTGC-3’ (P4).

At the age of 10 weeks, male APL-KO and WT were subjected to MI operation as previously described [18]. Briefly, after anaesthetization (medetomidine, midazolam, and butorphanol at dose of 0.15, 2.0, and 2.5mg/kg, i.p., respectively) and intubation, the left anterior descending coronary artery was permanently ligated with 8–0 nylon suture. At the indicated time points after sham or MI operation, mice were killed after anesthesia. Adenoviral vectors expressing APL or β-gal (1×10^9^ plaque-forming units/mouse) was intravenously injected into mice at 7 days after MI surgery.

### Histological analyses

Mice were sacrificed at 1 week or 4 weeks after permanent LAD ligation. Heart tissues samples were embedded in OCT compound (Sakura Finetek USA, Inc) and snap-frozen in liquid nitrogen. Tissue slices (5μm in thickness) were histologically analyzed. Heart sections were stained with Masson’s trichrome to detect infarct areas. The extent of infarct size was calculated as total infarct circumference divided by total left ventricular (LV) circumference or fibrosis area divided by total LV area [18,19]. Cardiomyocyte apoptosis in the remote zone and the border zone from infarct hearts was assessed by a terminal deoxynucleotidyl transferase-mediated dUTP-nick end labeling (TUNEL) staining using the In Situ Cell Death detection kit (Roche Diagnostics) as described previously [20,21]. Cryo-sections (5μm thickness) were fixed with 4% paraformaldehyde in PBS, permeabilized with 0.1% Triton X-100. DAPI was used for counter staining. Cardiomyocytes were determined by staining with sarcomeric actinin. The mean number of TUNEL-positive cells were counted in randomly selected fields of the slide.

### Echocardiographic analysis

Transthoracic echocardiography (Vevo 1100 imaging system (FUJIFILM Visual Sonics, Inc, Toronto, ON, Canada)) was performed to evaluate cardiac function of mice at 1 and 4 weeks after MI [18,19]. Left ventricular end-diastolic dimension (LVDd) and LV systolic dimension (LVSd) were measured by M-mode images. LV fractional shortening (FS) was calculated as (LVDd-LVSd)/LVDd×100 (%).

### Preparation of recombinant mouse adipolin protein

HEK293F cells were transfected using the Targefect-293F reagent with the pCDNA3.1 vector expressing a full-length mouse adipolin cDNA tagged with His according to the manufacturer’s instructions (Targeting Systems, El Cajon, CA, USA). HEK293F cells were cultured in Freestyle293 expression medium (12338; Life Technologies, Carlsbad, CA, USA) under 8% carbon dioxide. The culture supernatants were collected and purified using His Ni-NTA resin. Adipolin protein was eluted by incubation with imidazole and dialyzed with phosphate-buffered saline (PBS) [16].

### Cell culture

Primary cultures of neonatal rat ventricular myocytes (passage 1) were incubated in Dulbecco’s modified Eagle’s medium (DMEM) / F-12 supplemented with 10% Fetal Bovine Serum (FBS) as previously described [20–22]. After 16 hours serum starvation, cardiac myocytes were treated with adipolin protein at 300ng/ml or vehicle for the indicated length of time. In some experiments, cells were exposed to 16 hours hypoxia. Hypoxic conditions were generated using an AnaeroPack (Mitsubishi GAS Chemical). In some experiments, cardiac myocytes were pretreated with adipolin protein or vehicle for 60 minutes followed by stimulation with LPS for 6 hours. In some experiments, cardiac myocytes were pretreated with LY294002 (10μM) or vehicle for 60 minutes.

### Quantification of mRNA levels

Gene expression levels were analyzed by quantitative real-time PCR method. Total RNA was extracted from cultured cardiac myocytes and cell using RNeasy Mini Kit (Qiagen, Hilden, Germany). cDNA was prepared using a ReverTra Ace kit (TOYOBO, Osaka, Japan). Real-time PCR procedure was performed with a Bio-Rad real-time PCR detection system (Bio-Rad, Hercules, CA, USA) using the THUNDERBIRD SYBR qPCR Mix (TOYOBO, Osaka, Japan) as a double-standard DNA-specific dye [16,21]. The primers were listed in S1 Table. Expression levels of examined transcripts were divided by the corresponding level of β-actin expression and were expressed relative to the control group.

### Western blot analysis

Heart tissue or cell samples were homogenized in lysis buffer containing 20mM Tris-HCl (pH8.0), 150mM NaCl, 1mM Na2EDTA, 1mM EGTA, 1% Triton, 2.5mM sodium pyrophosphate, 1mM sodium orthovanadate, 1mM β-glycerophosphate, 1mM PMSF, leupeptin (1μg/mL), and Complete Mini Proteinase Inhibitor Cocktail (Roche). The protein concentration was calculated using a BCA protein assay kit (Thermo Scientific). Equal amounts of protein or serum were separated by denaturing SDS-PAGE and transferred onto PVDF membranes. Membranes were incubated with the primary antibodies, followed by incubation with the HRP-conjugated secondary antibodies. ECL prime system (GE Healthcare) was used for detection of the protein signal. The protein expression levels were determined by measurement of the band intensities using Image J software (National Institute of Health, USA) [23].

### Statistical analysis

Data are shown as mean ± S.E. The differences between two groups for variables with normal distributions were evaluated by unpaired Student’s t-test (Welch’s t-test was applied for variables with unequal variances). Differences between three or more groups were evaluated using one-way analysis of variance, with a post-hoc Tukey’s test. The differences between groups for variables with non-normal distribution were analyzed by Mann-Whitney U test (for two groups) or Steel-Dwass test (for three or more groups). Data distributions were evaluated by Shapiro-Wilk test. A P value < 0.05 denoted the presence of a statistically significant difference. All statistical calculations were performed by using JMP Pro 14 software (SAS Institute Inc., Cary, NC, USA).

## Results

### APL-KO mice exhibit exacerbated cardiac dysfunction after MI

WT and APL-KO mice were subjected to the permanent LAD ligation to create MI. There were no differences in body weight (BW) and heart rate in WT and APL-KO mice at 4 weeks after sham or MI operation (Table 1). Systolic BP was significantly lower in MI-operated WT mice than in sham-operated WT mice (Table 1). There were no significant differences in systolic BP between WT and APL-KO mice after MI surgery. The ratios of heart weight (HW) / body weight (BW) and lung weight (LW) / BW were significantly higher in APL-KO mice than in WT mice after MI operation (Table 1). On the other hand, no significant differences were observed in systolic BP, heart rate, HW and LW between WT and APL-KO mice after sham operation (Table 1). The survival rate was not different between APL-KO mice and WT mice after MI operation (S1 Fig in S1 File).

**Table 1:** Characteristics of WT and APL-KO mice at 4 weeks after sham or MI operation.

	Sham		MI	
	WT	APL-KO	WT	APL-KO
Body weight (g)	26.8 ± 0.6	26.0 ± 0.5	25.9 ± 0.2	26.5 ± 0.8
Systolic BP(mmHg)	108.3 ± 1.9	106.6 ± 2.4	96.6 ± 1.7**	97.5 ± 2.7
Heart rate (rpm)	605 ± 9	581 ± 17	621 ± 9	604 ± 9
Heart rate (rpm)	107 ± 3	107 ± 3	159 ± 5**	213 ± 8^##^
Heart rate (rpm)	147 ± 2	136 ± 4	195 ± 10*	246 ± 13^##^
HW/BW (mg/g)	3.99 ± 0.08	4.13 ± 0.06	6.16 ± 0.22**	8.05 ± 0.27^##^
LW/BW (mg/g)	5.48 ± 0.11	5.21 ± 0.11	7.55 ± 0.37**	9.45 ± 0.73^##^

Data are presented as mean ± S.E.

MI; myocardial infarction, BP; blood pressure.

N = 8 in Sham/WT group, N = 9 in Sham/APL-KO group, N = 19 in MI/WT group and N = 14 in MI/APL-KO group.

**P<0.01 for Sham/WT group

*P<0.05 for Sham/WT group

^##^P<0.01 for MI/WT group.

**Fig 1:**
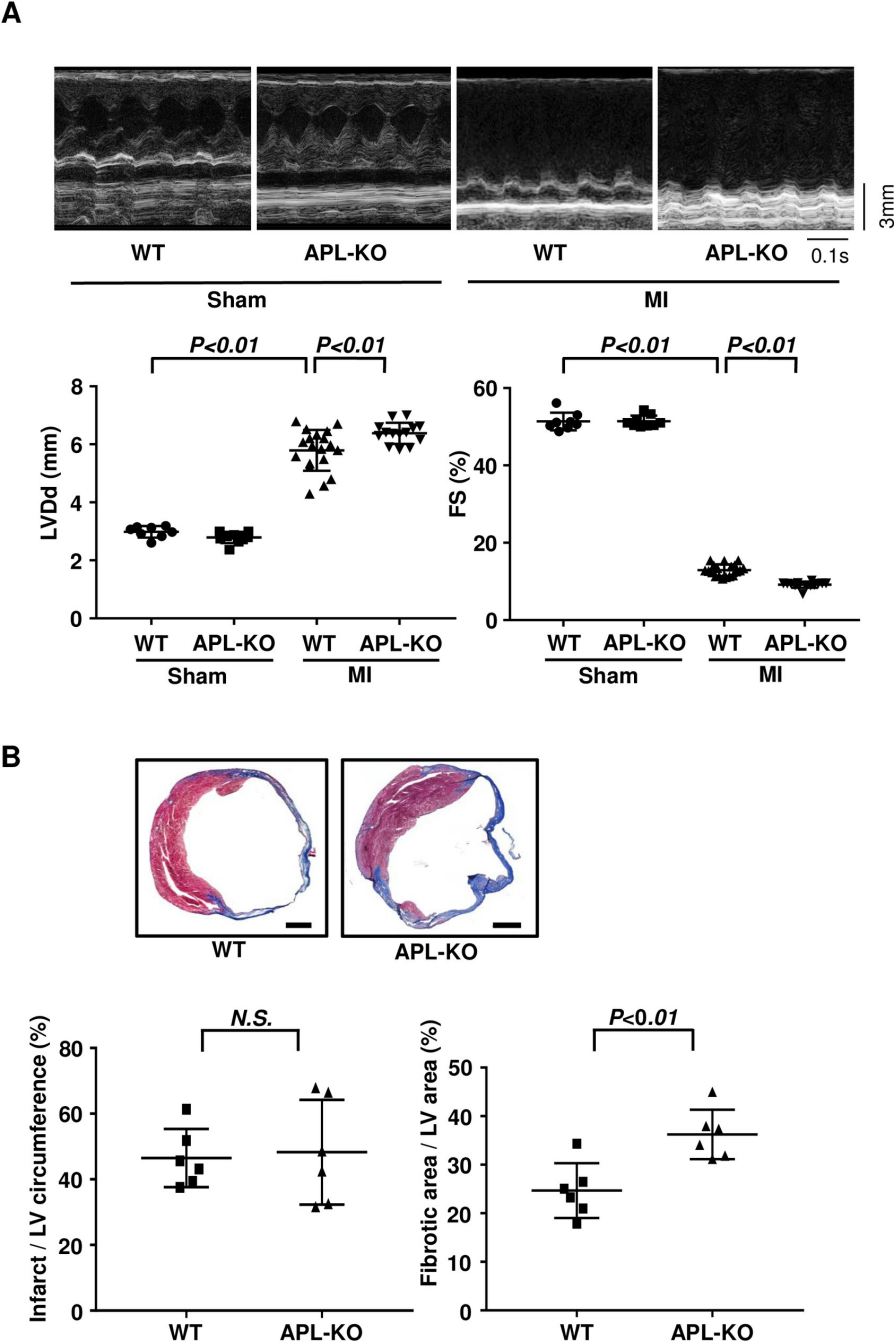
APL-KO mice exhibit enhanced LV dilatation and contractile dysfunction after MI. **(A)** Echocardiographic analyses of WT and APL-KO mice at 4 weeks after sham or MI operation. Left ventricular diastolic diameter (LVDd) and fractional shortening (FS) were analyzed. N = 8 in Sham/WT group. N = 9 in Sham/APL-KO group. N = 19 in MI/WT group. N = 14 in MI/APL-KO group. **(B)** Myocardial infarct size of WT or APL-KO mice at 4 weeks after MI. Upper panels show representative images of heart sections stained with Masson’s trichrome. Lower left panel shows quantitative analysis of percentage of total infarct circumference divided by total LV circumference. Lower right panel shows quantitative analysis of percentage of fibrotic area divided by total LV area. N = 6 in each group. Scale bars, 1 mm.

Echocardiographic analysis was performed to assess the cardiac dilatation and function in WT and APL-KO mice at 4 weeks after MI or sham operation. APL-KO mice showed increased LVDd, left ventricular end-diastolic volume (LVEDV), left ventricular end-systolic volume (LVESV) and decreased FS after MI compared with WT mice, whereas there were no significant differences in LVDd, LVEDV, LVESV and FS between WT and APL-KO mice after sham operation (Fig 1A, Table 2).

**Table 2:** Echocardiographic data of WT and APL-KO mice at 4 weeks after MI.

	Sham		MI	
	WT	APL-KO	WT	APL-KO
IVS (mm)	0.91 ± 0.02	0.90 ± 0.01	0.22 ± 0.01**	0.16 ± 0.01^##^
PW (mm)	0.89 ± 0.02	0.90 ± 0.02	0.79 ± 0.02*	0.77 ± 0.01
LVDd (mm)	2.98 ± 0.07	2.79 ± 0.06	5.79 ± 0.16**	6.38 ± 0.10^##^
FS (%)	51.4 ± 0.8	51.4 ± 0.5	12.9 ± 0.3**	9.2 ± 0.2^##^
LVEDV(μL)	34.7 ± 1.9	29.4 ± 1.6	169.3 ± 10.2**	208.0 ± 7.2^##^
LVESV(μL)	5.6 ± 0.4	4.7 ± 0.3	124.0 ± 8.3**	167.3 ± 6.5^##^

Data are presented as mean ± S.E.

MI; myocardial infarction, IVS; interventricular septum thickness, PW; posterior wall thickness, LVDd; left ventricular end-diastolic dimension, FS; fractional shortening, LVEDV; left ventricular end-diastolic volume, LVESV; left ventricular end-systolic volume.

N = 8 in Sham/WT group. N = 9 in Sham/APL-KO group. N = 19 in MI/WT group. N = 14 in MI/APL-KO group.

*P<0.05 for Sham/WT group

**P<0.01 for Sham/WT group

^##^P<0.01 for MI/WT group.

To evaluate the extent of myocardial infarct size after chronic ischemia, heart tissues were stained with Masson's trichrome. While APL-KO mice showed increased fibrotic infarct area at 4 weeks after MI operation compared with WT mice, there were no significant differences in the ratio of total infarct length to total LV circumstance between WT and APL-KO mice (Fig 1B).

### APL-KO mice exhibit increased inflammatory response and apoptosis in post-MI hearts

**Fig 2:**
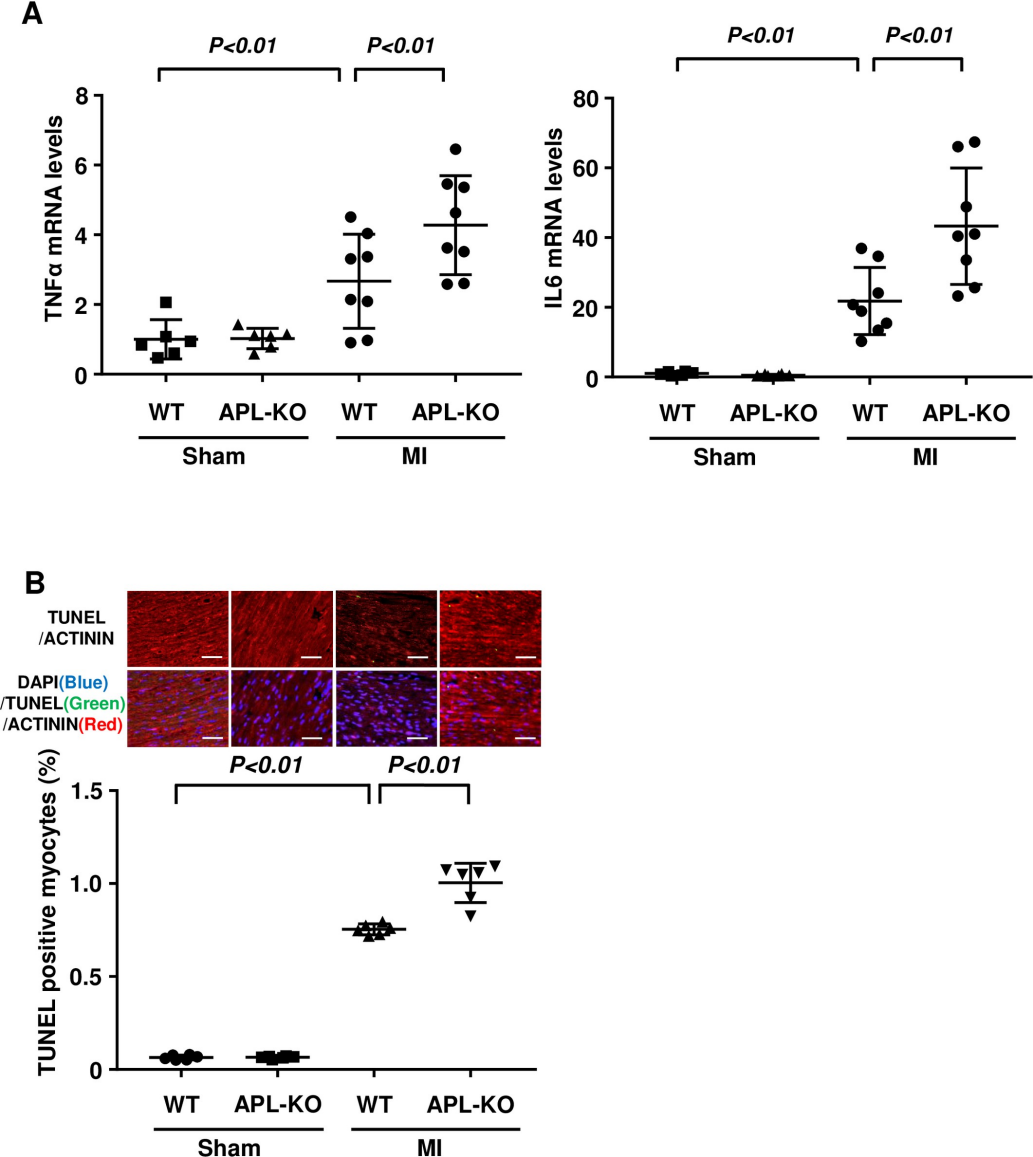
APL-KO mice show enhanced inflammatory response and apoptosis in the post-MI heart. (A) The mRNA levels of TNFα and IL6 in the heart tissues from WT and APL-KO mice at 7 days after sham or MI operation. N = 6 in Sham groups. N = 8 in MI groups. (B) Cardiomyocyte apoptosis in the hearts of WT and APL-KO mice at 4 weeks after sham or MI operation. Upper panels show representative photographs of heart sections stained with TUNEL (green), sarcomeric actinin (red) and DAPI (blue). Lower panel shows quantitative analysis of TUNEL-positive cardiomyocytes. N = 6 in each group. Scale bars, 50 μm.

Enhanced inflammatory response contributes to the development of adverse cardiac remodeling after MI [24]. To determine the inflammatory response in the hearts at 7 days after MI or sham operation, proinflammatory cytokine expression was quantified by real-time PCR. The mRNA levels of tumor necrosis factor α (TNFα) and interleukin 6 (IL6) were increased in APL-KO mice compared with WT mice after MI operation, whereas no differences were observed in TNFα and IL6 expression between WT and APL-KO mice after sham operation (Fig 2A). MI surgery significantly reduced the expression of adipolin in the hearts of WT mice compared with sham operation (S2 Fig in S1 File).

Cardiomyocyte apoptosis plays an important role during the progression of heart failure after MI [25]. The extent of cardiomyocyte apoptosis at the remote and the border zone of infarct hearts was evaluated by TUNEL staining. APL-KO mice showed increased numbers of TUNEL-positive cardiomyocytes at the remote and the border zone after MI compared with WT mice (Fig 2B, S3 Fig in S1 File), whereas little or no TUNEL-positive cells were detected in the hearts of WT and APL-KO mice after sham operation (Fig 2B).

### Adipolin reduces cardiomyocyte inflammatory response and apoptosis

**Fig 3:**
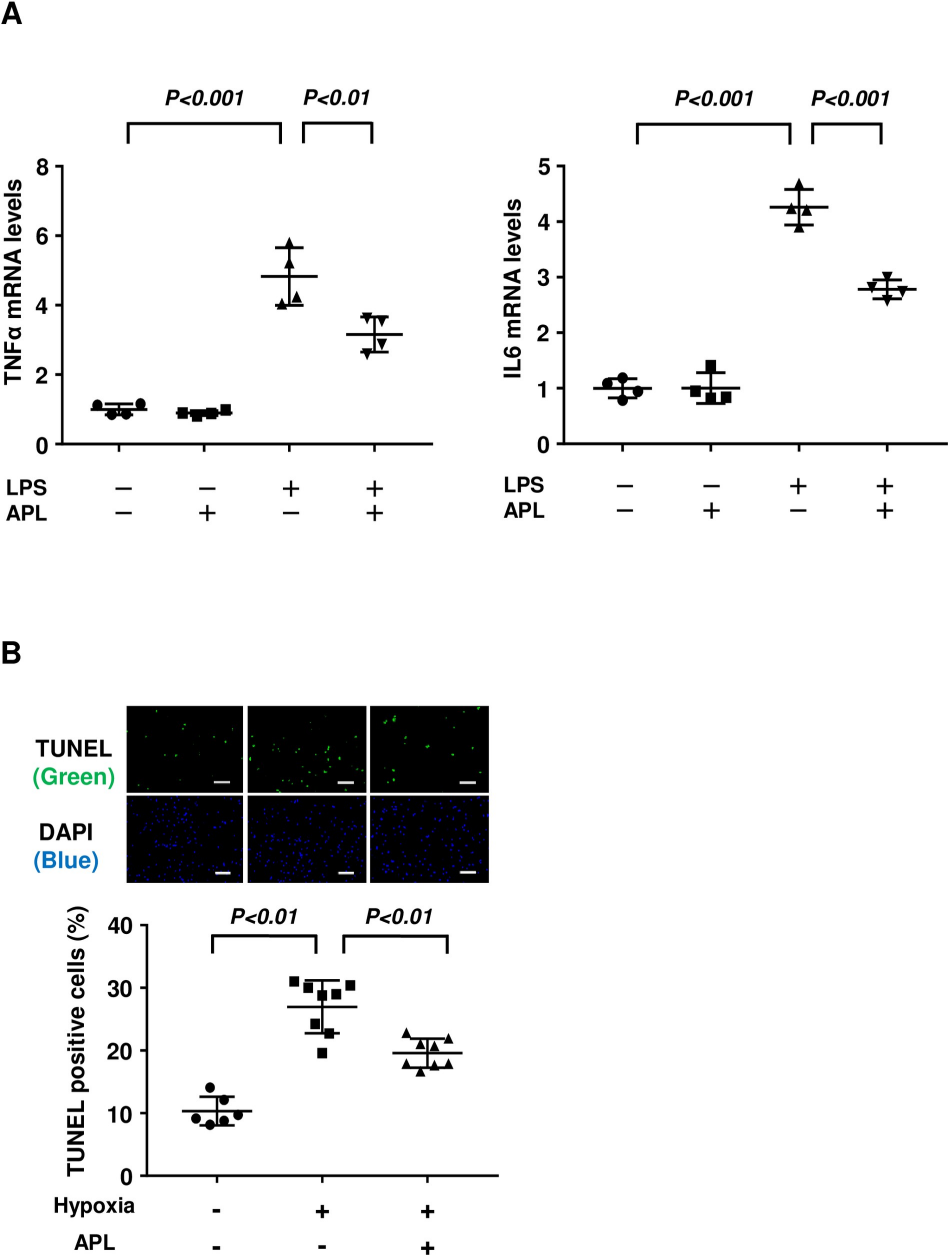
Adipolin reduces inflammatory response and apoptosis in cardiomyocytes. **(A)** Effect of adipolin on inflammatory response to LPS in cardiomyocytes. NRVMs were pretreated with adipolin protein (300 ng/ml) or vehicle for 60 minutes followed by stimulation with LPS (100 ng/ml) or vehicle for 6 hours. The mRNA expression of TNFα and IL6 was measured by RT-PCR method and expressed relative to β-actin levels N = 4 in each group. **(B)** Effect of adipolin on cardiomyocyte apoptosis induced by hypoxia. Neonatal rat ventricular myocytes (NRVMs) were treated with adipolin protein (300 ng/ml) or vehicle under conditions of normoxia or hypoxia for 16 hours. NRVMs were stained with TUNEL (green) and DAPI (blue), and quantitative analysis of TUNEL-positive myocytes was performed. N = 6 in a normoxia group. N = 8 in hypoxia groups. Scale bars, 100 μm.

To investigate the effects of adipolin on inflammatory response at the cellular level, neonatal rat ventricular myocytes (NRVMs) were cultured in the presence or absence of recombinant adipolin protein followed by stimulation with lipopolysaccharide (LPS) or vehicle. Pretreatment of NRVMs with adipolin protein attenuated LPS-stimulated expression of TNFα and IL6 (Fig 3A).

To test the effect of adipolin on cardiomyocyte apoptosis, NRVMs were cultured in the presence or absence of adipolin protein under conditions of normoxia or hypoxia, and subjected to TUNEL staining. Hypoxic conditions resulted in an increased frequency of TUNEL-positive NRVMs, which was suppressed by treatment with adipolin protein (Fig 3B). These data indicated that adipolin can act as an anti-inflammatory and anti-apoptotic factor.

### Adipolin reduces inflammatory response and apoptosis of cardiomyocytes through the PI3 kinase/Akt signaling pathway

**Fig 4:**
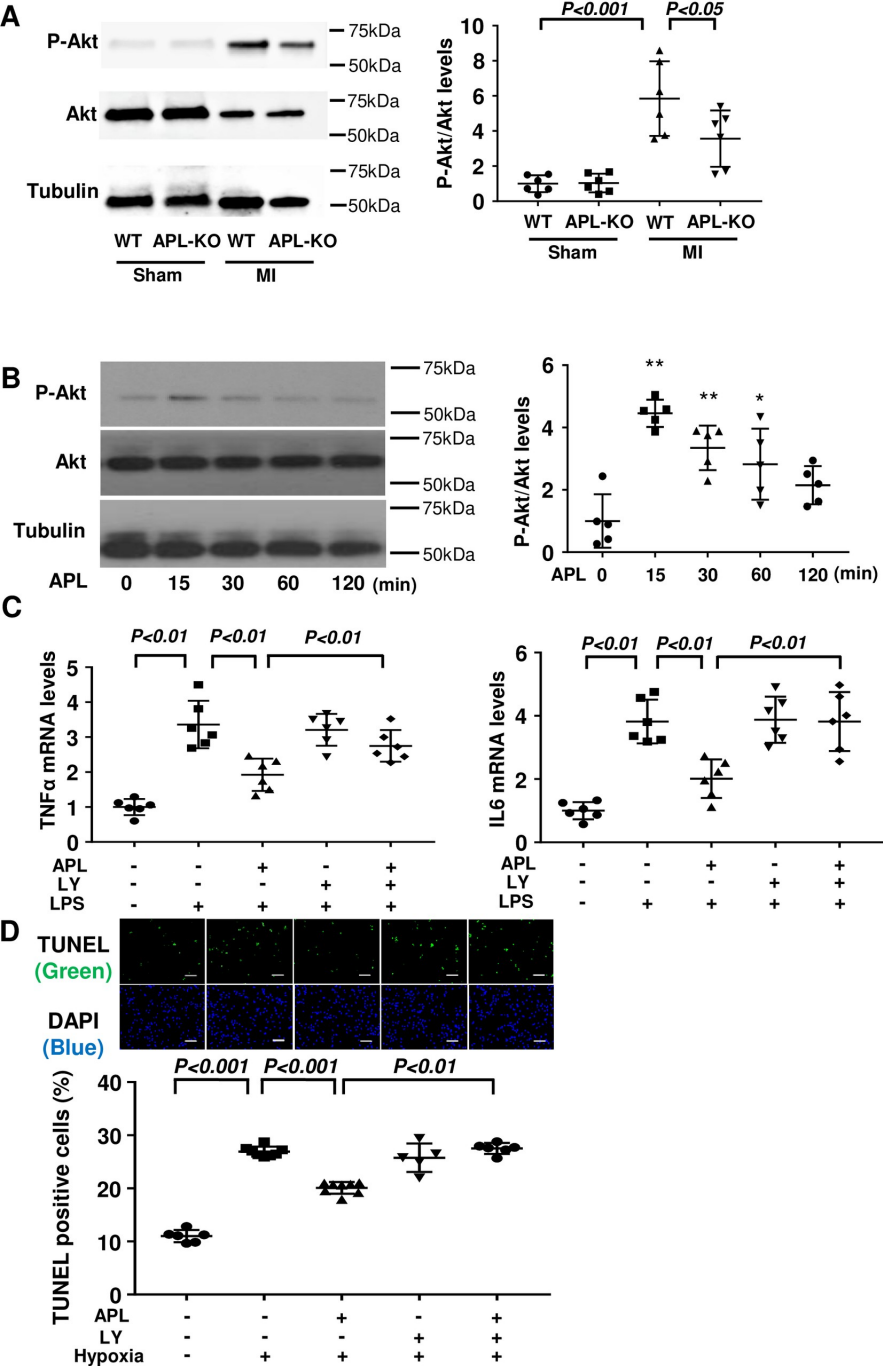
Adipolin attenuates inflammatory response and apoptosis of cardiomyocytes through the PI3 kinase/Akt signaling pathway. **(A)** Western blot analysis of phosphorylated Akt (P-Akt), Akt, and α-tubulin (Tubulin) in heart tissues from WT and APL-KO mice at 7 days after MI. Right panels show quantitative analyses of P-Akt levels relative to Akt as evaluated by Image J program. N = 6 in each group. **(B)** Time course changes in phosphorylation of Akt after adipolin treatment as assessed by Western blot analysis. Neonatal rat ventricular myocytes (NRVMs) were treated with adipolin protein (300 ng/ml) or vehicle for indicated lengths of time. Right panels show the quantitative analyses of P-Akt levels relative to Akt as evaluated by Image J program. N = 5 in each group. *P<0.05 for 0 min. **P<0.01 for 0 min. **(C)** Involvement of Akt in adipolin-mediated inhibition of LPS-induced expression of TNFα and IL6 in NRVMs. NRVMs were pretreated with PI3-kinase inhibitor (LY294002)(10 μM) or vehicle for 60 minutes, and treated with adipolin protein (300 ng/ml) or vehicle for 60 minutes followed by stimulation with LPS (100 ng/ml) or vehicle for 6 hours. N = 6 in each group. **(D)** Involvement of Akt in adipolin-mediated inhibition of NRVM apoptosis. NRVMs were pretreated with PI3-kinase inhibitor (LY294002)(10 μM) or vehicle for 60 minutes followed by treatment with adipolin protein (300 ng/ml) or vehicle for 16 hours under conditions of normoxia or hypoxia. NRVMs were stained with TUNEL (green) and DAPI (blue), and quantitative analysis of TUNEL-positive myocytes was performed. N = 6 in normoxia and LY294002 groups. N = 8 in hypoxia groups. Scale bars, 100 μm.

Because Akt is a mediator for metabolic and vascular function of adipolin [15,16], we assessed phosphorylation levels of Akt in the hearts of WT and APL-KO mice after MI. APL-KO mice showed decreased phosphorylation of Akt in the post-MI hearts compared with WT mice (Fig 4A). There were no differences in phosphorylation levels of Akt between WT and APL-KO mice after sham operation. Thus, it is plausible that Akt can mediate the protective actions of adipolin on cardiac remodeling after MI.

We also assessed the phosphorylation levels of Akt in NRVMs by Western blot analysis. Treatment of NRVMs with adipolin protein increased the phosphorylation of Akt with maximal levels occurring at 15 minutes (Fig 4B). To test whether Akt participates in anti-inflammatory effects of adipolin, NRVMs were pretreated with the PI3-kinase inhibitor LY294002 or vehicle followed by stimulation with adipolin protein or vehicle. Pretreatment with LY294002 reversed the inhibitory effects of adipolin on LPS-induced expression of TNFα and IL6 in NRVMs (Fig 4C). Furthermore, pretreatment with LY294002 canceled adipolin-mediated inhibition of NRVM apoptosis under conditions of hypoxia (Fig 4D). Collectively, these data suggest that adipolin can attenuate inflammatory response and apoptosis of cardiomyocytes, at least in part, through the Akt-dependent signaling pathway.

### Systemic administration of adipolin after MI ameliorates cardiac function and remodeling

**Fig 5:**
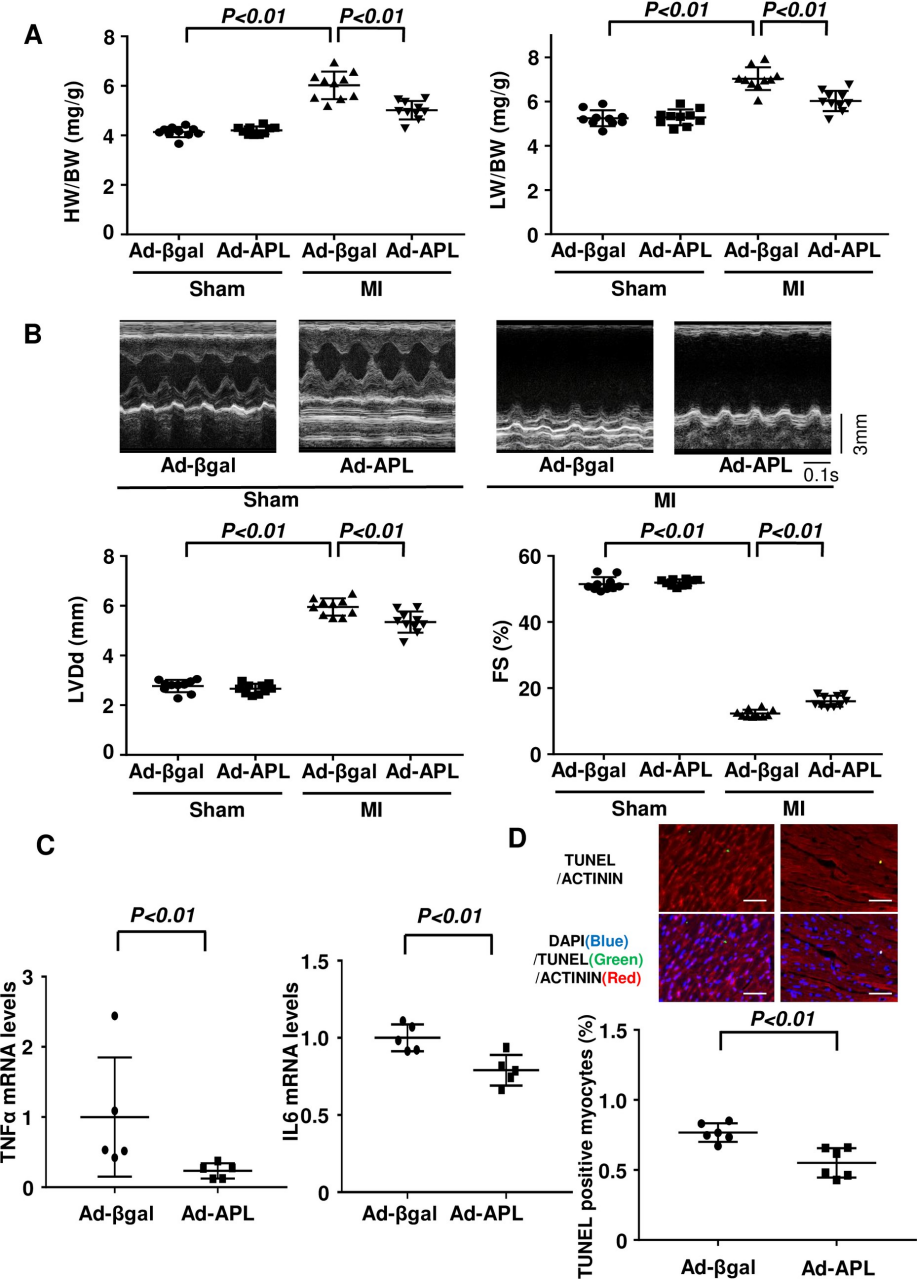
Systemic administration of adipolin ameliorates cardiac function after MI. **(A)** Left panel shows heart weight (HW)/body weight (BW) ratio in Ad-βgal- and Ad-APL-treated WT mice at 4 weeks after sham and MI operation. N = 10 in each group. Right panel shows lung weight (LW)/BW ratio in Ad-βgal- and Ad-APL-treated WT mice at 4 weeks after sham and MI operation. N = 10 in each group. **(B)** Echocardiographic analyses of Ad-βgal- or Ad-APL-treated WT mice at 4 weeks after sham and MI operation. Left ventricular diastolic diameter (LVDd) and fractional shortening (FS) were analyzed. N = 10 in each group. **(C)** The mRNA levels of TNFα and IL6 in the heart tissues from Ad-βgal- or Ad-APL-treated WT mice at 4 weeks after MI. N = 5 in each group. **(D)** Cardiomyocyte apoptosis in the hearts of Ad-βgal- or Ad-APL-treated WT mice at 4 weeks after MI operation. Upper panels show representative photos of heart sections stained with TUNEL (green), sarcomeric actinin (red) and DAPI (blue). Lower panel shows quantitative analysis of TUNEL-positive cardiomyocytes. N = 6 in each group. Scale bars, 50 μm.

Finally, to investigate whether increased plasma adipolin levels after MI affect cardiac function and remodeling, we systemically administered adenoviral vectors expressing adipolin (Ad-APL) or β-gal (Ad-β-gal, control) to WT mice at 7 days after sham or MI operation. Ad-APL-treated mice showed 2.15 ± 0.32 fold increase in plasma APL levels at 7 days after intravenously injection i.e. at 2 weeks after MI, as assessed by Western blot analysis (N = 8 in each group, P<0.01). In contrast, there were no differences in adipolin expression of MI hearts between Ad-APL-treated and Ad-βgal-treated mice (S4 Fig in S1 File). HW/BW and LW/BW ratios were significantly lower in Ad-APL-treated mice than in control mice at 4 weeks after MI operation, whereas no significant differences were observed in HW/BW and LW/BW between Ad-β-gal-treated and Ad-APL-treated WT mice after sham operation (Fig 5A). Echocardiographic analysis revealed that Ad-APL-treated mice exhibited decreased LVDd, LVEDV, LVESV and increased FS at 4 weeks after MI operation compared with control mice, whereas there were no significant differences in LVDd, LVEDV, LVESV and FS between Ad-β-gal-treated and Ad-APL-treated WT mice after sham operation (Fig 5B, S2 Table). Ad-APL treatment significantly suppressed expression of TNFα and IL6 in the post-MI hearts of WT mice (Fig 5C). Ad-APL treatment also reduced apoptosis at the remote and the border zone of infarct hearts in WT mice (Fig 5D, S5 Fig in S1 File).

## Discussion

Our present study provides the first evidence that adipolin protects against adverse ventricular remodeling following MI. APL-KO mice exhibited exacerbation of LV contractile dysfunction after MI. APL-KO mice also exhibited increased expression of inflammatory mediators and increased cardiomyocyte apoptosis in post-MI hearts. Systemic adipolin administration to WT mice after MI improved cardiac dysfunction, inflammatory response and apoptosis. Treatment of cultured cardiomyocytes with adipolin protein reduced LPS-stimulated expression of inflammatory mediators and hypoxia-induced apoptosis. These data indicated that adipolin can ameliorate pathological remodeling of myocardium after permanent ischemia, at least in part, through reduction of inflammatory response and apoptosis in cardiomyocytes.

Enhanced inflammatory response contributes to exacerbation of post-infarct myocardial remodeling [24,26,27]. Administration of TNFα inhibitor improves adverse cardiac remodeling after MI [28]. In addition, TNFα receptor 1 deficient mice show ameliorated LV function and hypertrophy post-MI [29]. Our data demonstrated that adipolin negatively regulated expression of pro-inflammatory mediators including TNFα in the post-MI hearts. Furthermore, adipolin suppressed LPS-induced expression of pro-inflammatory mediators such as TNFα in cultured cardiomyocytes. Therefore, it is likely that adipolin can improve post-MI cardiac remodeling, at least in part, by inhibiting cardiomyocyte inflammatory response. We have previously shown that adipolin improves glucose intolerance in obesity through attenuation of inflammatory response in adipose tissue [14]. Recently, we have reported that adipolin attenuates pathological vascular remodeling in response to mechanical injury through reduction of inflammatory response in vascular walls [16]. Collectively, these findings indicated that adipolin can serve as an anti-inflammatory adipokine that prevents the development of cardiometabolic disorders.

Cardiomyocyte apoptosis is a key feature of the pathological cardiac remodeling at the chronic phase after MI [25,30]. Our current data indicated that adipolin deficiency increased apoptosis in the post-MI hearts. Conversely, adipolin administration attenuated apoptosis in post-MI hearts of mice. In addition, treatment of cultured cardiomyocytes with adipolin reduced apoptosis under hypoxic conditions. Thus, it is conceivable that adipolin functions as an anti-apoptotic factor. Our in vitro data also indicated that adipolin reduced hypoxia-induced apoptosis of cardiomyocytes through the Akt-dependent pathway. Our in vivo data showed that adipolin deficiency led to reduced phosphorylation of Akt in post-MI hearts. Akt activation has been reported to attenuate cardiomyocyte apoptosis and cardiac ischemic injury [31]. Taken together, these findings suggest that adipolin can ameliorate adverse cardiac remodeling following MI, at least in part, by its ability to reduce cardiomyocyte apoptosis through the Akt-dependent mechanism.

It has been shown that Akt reduces inflammatory response in various cells including cardiomyocytes [21,32,33]. Our data demonstrated that adipolin reduced inflammatory response to LPS in cardiomyocytes through the Akt-dependent pathway. It has been reported that adipolin improves glucose metabolism through activation of Akt in adipose tissue and liver [15]. Thus, Akt may be a crucial mediator of adipolin function.

Adipolin expression is regulated by transcriptional factors, Krϋppel like factor (KLF) 3 and KLF15 [17,34]. KLF3 negatively regulates adipolin expression [35]. KLF3 deficient mice are resistant to diet-induced obesity and glucose intolerance with an accompanying increase in adipolin expression [35]. In contrast, KLF15 positively regulates adipolin expression in adipocytes [36]. KLF15 expression is reduced in adipose tissue in obese mice. TNFα treatment reduces mRNA levels of KLF15 and adipolin in cultured adipocytes. Thus, it is likely that adipose tissue inflammation in obesity contributes to reduction of adipolin expression partly through down-regulation of KLF15. These data indicate that adipolin may be down-regulated by metabolically unhealthy obesity. It has been shown that obese mice fed a diet supplemented with a polyphenol-rich plant extract induces healthy adiposity and extended lifespan [37]. Expression profiles of adipokines including adiponectin are reported to differ between metabolically unhealthy and healthy obese individuals [38]. Since the relationship of adipolin expression levels with healthy obese phenotypes has not been clarified, this requires future investigation.

In conclusion, we demonstrated that adipolin prevents post-MI cardiac remodeling in vivo by its ability to reduce myocyte apoptosis and inflammatory response. We also found that an increased level of circulating adipolin contributes to improvement of cardiac function and remodeling after MI. We have recently shown that adipolin attenuates the adverse remodeling of vascular wall after injury in vivo [16]. It has been reported that circulating adipolin levels are decreased in patients with coronary heart disease [39]. Thus, these findings indicated that therapeutic approaches to increase adipolin production or enhance adipolin signaling pathways could be valuable for prevention or treatment of cardiovascular diseases.

## Supporting information

S1 Table Primers used for quantitative RT-PCR. (DOCX)

S2 Table Echocardiographic data of WT mice treated Ad-βgal or Ad-APL at 4 weeks after MI. (DOCX)

S1 Raw images (PDF)

S1 File (PDF)
