# Supplementary material for: Adipolin/C1q/Tnf-related protein 12 prevents adverse cardiac remodeling after myocardial infarction
Source: PLoS One. 2020 Dec 4;15(12):e0243483. doi: 10.1371/journal.pone.0243483 (PMC7717554; doi:10.1371/journal.pone.0243483)
Supplement: S1 Table — (DOCX) [file pone.0243483.s001.docx]

**Supplemental Table 1.**

**Primers used for quantitative RT-PCR**

**Mouse**

β-actin: forward 5’-AGAGGGAAATCGTGCGTGAC-3’

reverse 5’-CAATAGTGATGACCTGGCCGT-3’

TNFα: forward 5’-CGGAGTCCGGGCAGGT-3’

reverse 5’-GCTGGGTAGAGAATGGATGAACA-3’

IL6: forward 5’-GCTACCAAACTGGATATAATCAGG-3’

reverse 5’-CCAGGTAGCTATGGTACTCCAGAA-3’

APL: forward 5’-CGATTGTGGATTGAGGAA-3’

reverse 5’-GTTTGGAGTTCTTTATTGCTAT-3’

**Rat**

β-actin: forward 5’-GGTCATCACTATCGGCAATG-3’

reverse 5’-AGGTCTTTACGGATGTCAAC-3’

TNFα: forward 5’-CCAATCTGTGTCCTTCTAAC-3’

reverse 5’-GTTTCTGAGCATCGTAGTTG-3’

IL6: forward 5’-TACCCCAACTTCCAATGCTC-3’

reverse 5’-TTGCCGAGTAGACCTCATAGTG-3’

TNFα; tumor necrosis factor α, IL6; interleukin 6, APL; adipolin.
