# Supplementary material for: Adipolin/C1q/Tnf-related protein 12 prevents adverse cardiac remodeling after myocardial infarction
Source: PLoS One. 2020 Dec 4;15(12):e0243483. doi: 10.1371/journal.pone.0243483 (PMC7717554; doi:10.1371/journal.pone.0243483)
Supplement: S2 Table — (DOCX) [file pone.0243483.s002.docx]

**Supplemental Table 2**

**Echocardiographic data of WT mice treated Ad-βgal or Ad-APL at 4 weeks after MI**

**Sham MI**

**Ad-βgal Ad-APL Ad-βgal Ad-APL**

IVS (mm) 0.97 ± 0.01 0.97 ± 0.01 0.21 ± 0.01** 0.24 ± 0.01^#^

PW (mm) 0.98 ± 0.01 0.98 ± 0.01 0.84 ± 0.02** 0.83 ± 0.02

LVDd (mm) 2.77 ± 0.08 2.66 ± 0.06 5.95 ± 0.11** 5.34 ± 0.14^##^

FS (%) 51.5 ± 0.7 51.9 ± 0.3 12.3 ± 0.4** 16.0 ± 0.5^##^

LVEDV(μL) 29.1 ± 1.9 26.3 ± 1.5 177.2 ± 7.4** 138.9 ± 7.9^##^

LVESV(μL) 4.6 ± 0.3 4.0 ± 0.3 131.2 ± 6.3** 92.5 ± 5.9^##^

Data are presented as mean ± S.E.

MI; myocardial infarction, IVS; interventricular septum thickness, PW; posterior wall thickness, LVDd; left ventricular end-diastolic dimension, FS; fractional shortening, LVEDV; left ventricular end-diastolic volume, LVESV; left ventricular end-systolic volume.

N=10 in Sham/ Ad-βgal group. N=10 in Sham/ Ad-APL group. N=10 in MI/ Ad-βgal group. N=10 in MI/Ad-APL group.

**P<0.01 for Sham/Ad-βgal group, ^#^P<0.05 for MI/Ad-βgal group, ^##^P<0.01 for MI/Ad-βgal group.
