# Supplementary figures and images for: Adipolin/C1q/Tnf-related protein 12 prevents adverse cardiac remodeling after myocardial infarction
Source: PLoS One. 2020 Dec 4;15(12):e0243483. doi: 10.1371/journal.pone.0243483 (PMC7717554; doi:10.1371/journal.pone.0243483)

Raw images

Figure 4A

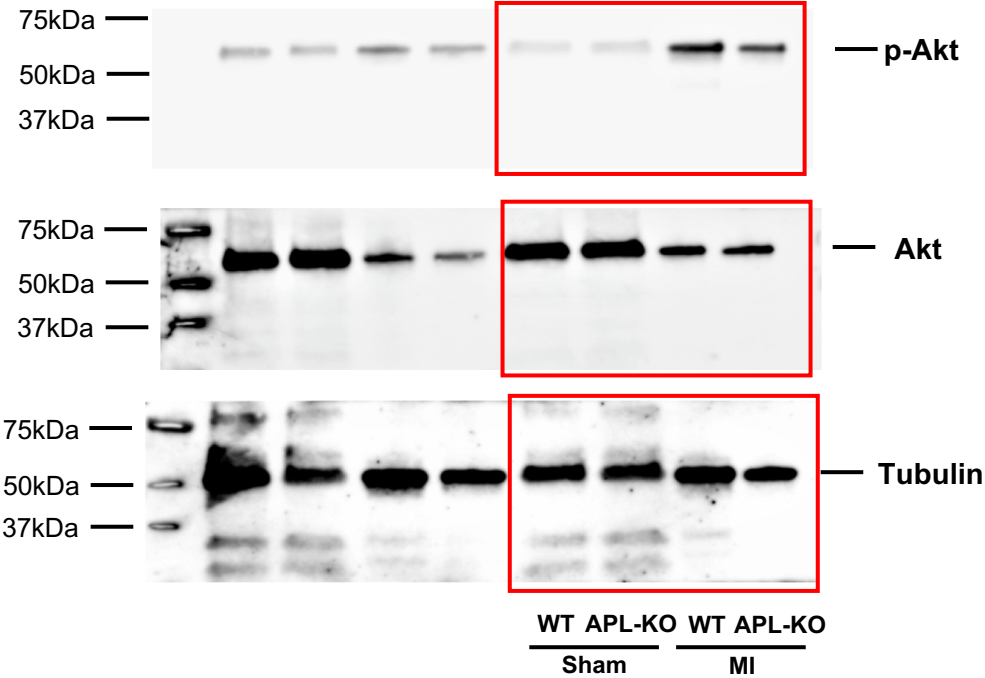

Figure 4B

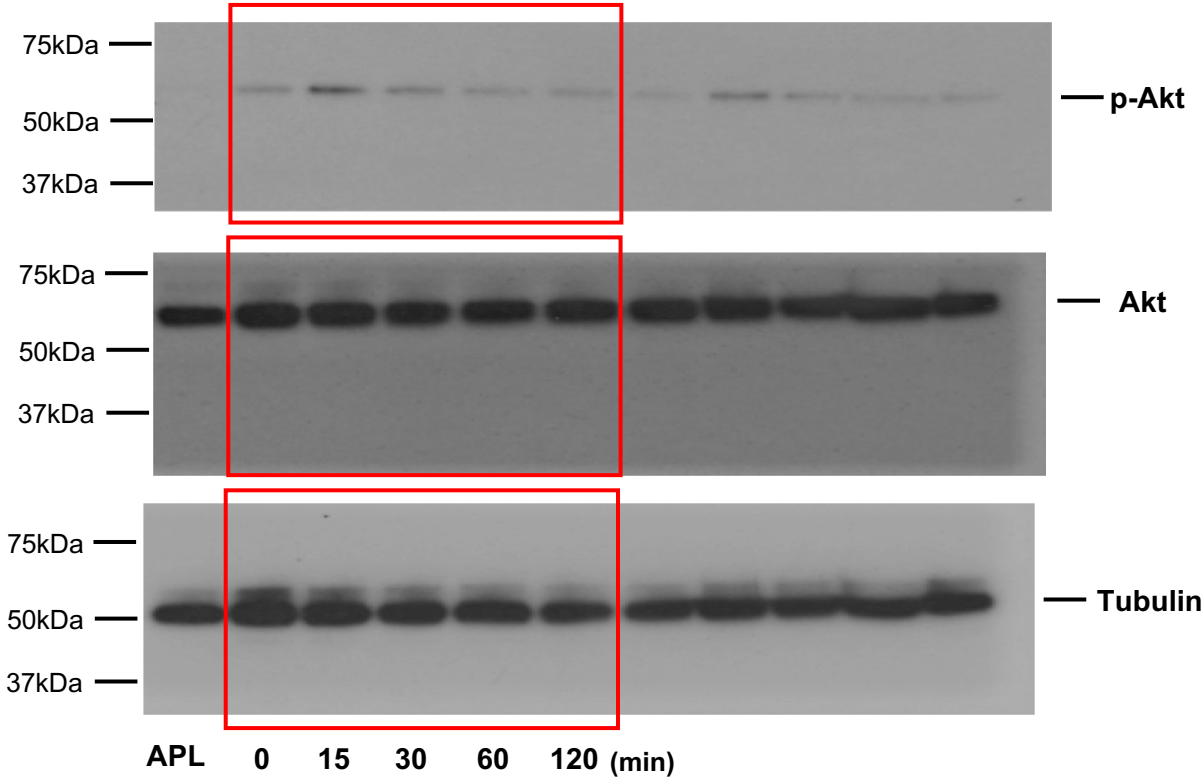

Supplement: S1 Raw images — (PDF) [file pone.0243483.s003.pdf]
