# Supplementary material for: Adipolin/C1q/Tnf-related protein 12 prevents adverse cardiac remodeling after myocardial infarction
Source: PLoS One. 2020 Dec 4;15(12):e0243483. doi: 10.1371/journal.pone.0243483 (PMC7717554; doi:10.1371/journal.pone.0243483)

Supplemental Figure legends

Supplemental Figure 1. Survival curves of APL-KO mice and WT mice after MI operation. Kaplan-Meier survival curves of WT and APL-KO mice for 28 days after MI operation. N=43 in MI/WT group. N=46 in MI/APL-KO group. (The blue line; MI/WT group, The red line; MI/APL-KO group).

Supplemental Figure 2. MI surgery significantly reduces the expression of adipolin in the hearts of WT mice. We assessed mRNA levels of APL in the heart tissues from WT mice at 7 days after sham or MI operation by RT-PCR method. N=5 in each group.

Supplemental Figure 3. APL deficiency increases cardiomyocyte apoptosis at border zone of post-MI hearts. We assessed cardiomyocyte apoptosis of peri infarct area in the hearts of WT and APL-KO mice at 4 weeks after MI operation. Upper panels show representative photographs of heart sections stained with TUNEL (green), sarcomeric actinin (red) and DAPI (blue). Lower panel shows quantitative analysis of TUNEL-positive cardiomyocytes. N=6 in each group. Scale bars, 50 μm.

Supplemental Figure 4. Systemic administration of APL has no effects on APL expression in post-MI hearts. We assessed mRNA levels of APL in the heart tissues from Ad-βgal-treated or Ad-APL-treated WT mice at 4 weeks after MI by RT-PCR method. N=5 in each group.

Supplemental Figure 5. Systemic administration of APL reduces cardiomyocyte apoptosis at border zone of post-MI hearts. We assessed cardiomyocyte apoptosis of peri infarct area in the hearts of Ad-βgal-treated or Ad-APL-treated WT mice at 4 weeks after MI operation. Upper panels show representative photos of heart sections stained with

TUNEL (green), sarcomeric actinin (red) and DAPI (blue). Lower panel shows quantitative analysis of TUNEL-positive cardiomyocytes. N=6 in each group. Scale bars, 50 μm.

Supplemental Figure 1

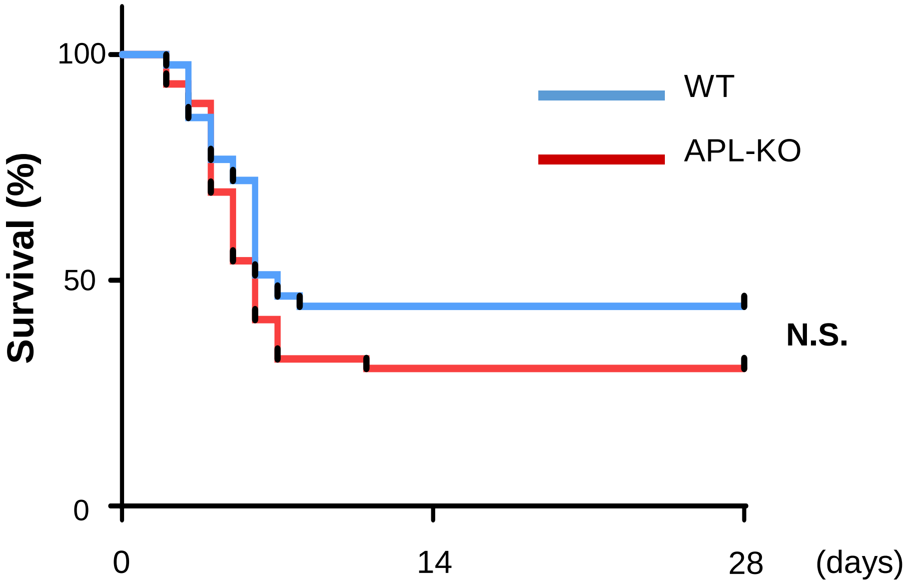

Supplemental Figure 2

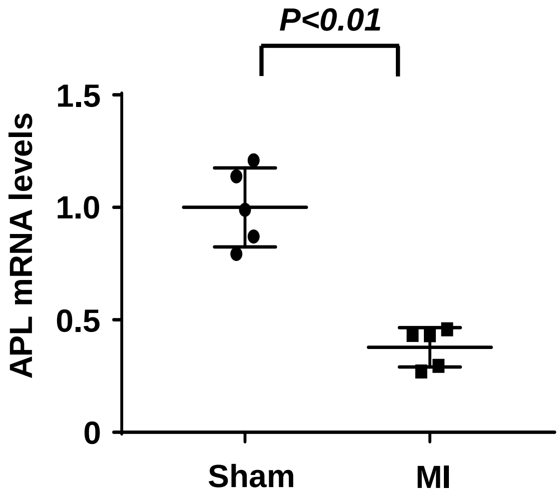

Supplemental Figure 3

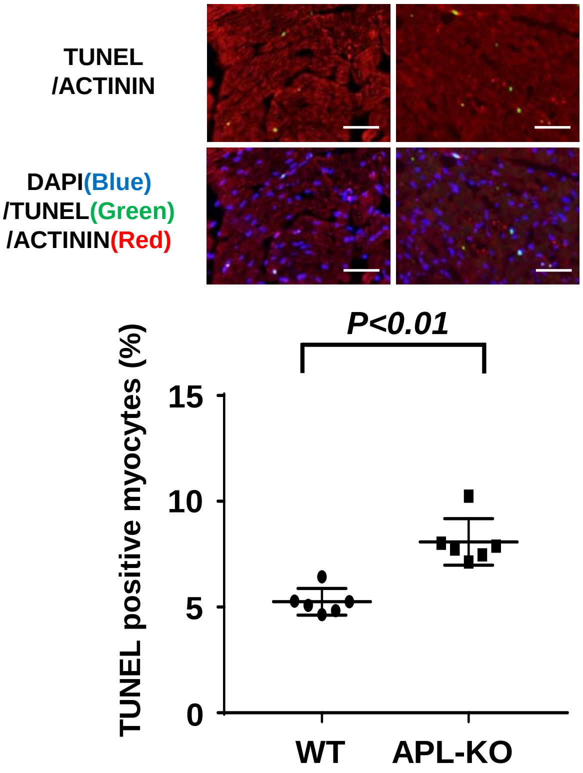

Supplemental Figure 4

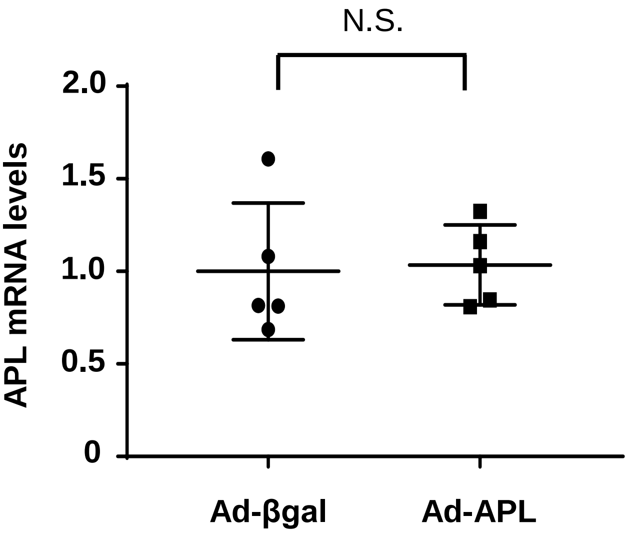

Supplemental Figure 5

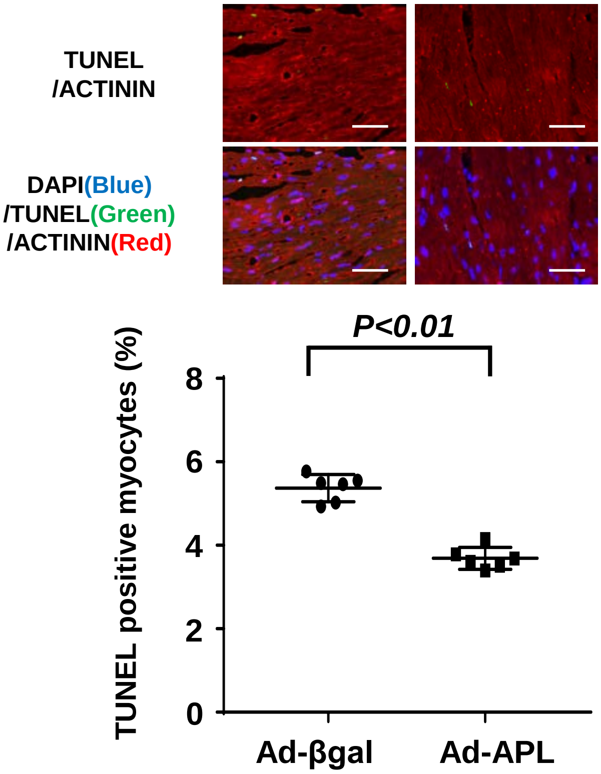

Supplement: S1 File — (PDF) [file pone.0243483.s004.pdf]
